# Type I Interferon-Independent Dendritic Cell Priming and Antitumor T Cell Activation Induced by a *Mycoplasma fermentans* Lipopeptide

**DOI:** 10.3389/fimmu.2018.00496

**Published:** 2018-03-14

**Authors:** Yohei Takeda, Masahiro Azuma, Kenji Funami, Hiroaki Shime, Misako Matsumoto, Tsukasa Seya

**Affiliations:** ^1^Department of Vaccine Immunology, Hokkaido University Graduate School of Medicine, Sapporo, Japan

**Keywords:** antitumor adjuvant, cross-presentation, diacylated lipopeptide, programmed death ligand-1 blockade, toll-like receptor 2

## Abstract

*Mycoplasma fermentans*-derived diacylated lipoprotein M161Ag (MALP404) is recognized by human/mouse toll-like receptor (TLR) 2/TLR6. Short proteolytic products including macrophage-activating lipopeptide 2 (MALP2) have been utilized as antitumor immune-enhancing adjuvants. We have chemically synthesized a short form of MALP2 named MALP2s (*S*-[2,3-bis(palmitoyloxy)propyl]-CGNNDE). MALP2 and MALP2s provoke natural killer (NK) cell activation *in vitro* but only poorly induce tumor regression using *in vivo* mouse models loading NK-sensitive tumors. Here, we identified the functional mechanism of MALP2s on dendritic cell (DC)-priming and cytotoxic T lymphocyte (CTL)-dependent tumor eradication using CTL-sensitive tumor-implant models EG7 and B16-OVA. Programmed death ligand-1 (PD-L1) blockade therapy in combination with MALP2s + ovalbumin (OVA) showed a significant additive effect on tumor growth suppression. MALP2s increased co-stimulators CD80/86 and CD40, which were totally MyD88-dependent, with no participation of toll-IL-1R homology domain-containing adaptor molecule-1 or type I interferon signaling in DC priming. MALP2s + OVA consequently augmented proliferation of OVA-specific CTLs in the spleen and at tumor sites. Chemokines and cytolytic factors were upregulated in the tumor. Strikingly, longer duration and reinvigoration of CTLs in spleen and tumors were accomplished by the addition of MALP2s + OVA to α-PD-L1 antibody (Ab) therapy compared to α-PD-L1 Ab monotherapy. Then, tumors regressed better in the MALP2s/OVA combination than in the α-PD-L1 Ab monotherapy. Hence, MALP2s/tumor-associated antigens combined with α-PD-L1 Ab is a good therapeutic strategy in some mouse models. Unfortunately, numerous patients are still resistant to PD-1/PD-L1 blockade, and good DC-priming adjuvants are desired. Cytokine toxicity by MALP2s remains to be settled, which should be improved by chemical modification in future studies.

## Introduction

Toll-like receptor (TLR) 2 is a pattern-recognition receptor (PRR) that recognizes microbial lipopeptides, lipoproteins, and peptidoglycans (1). We happened to identify the mycoplasma lipoprotein M161Ag, also called MALP404 (2), as a TLR2 agonist (3, 4). Notably, antigen-presenting dendritic cells (DCs) express TLR2 in addition to TLR3. TLR2, unlike TLR3, shows a broad expression spectrum including endothelial cells, epithelial cells, and immune cells (5, 6). Macrophage-activating lipopeptide 2 (MALP2) is a diacylated lipopeptide isolated from the outer membrane of *Mycoplasma fermentans* (7) and is known to be a proteolytic product of M161Ag (2–4). MALP2 is an agonistic ligand of the TLR2/6 heterodimer and induces inflammatory cytokine production from macrophages, monocytes, and DCs (8, 9). MALP2, as well as a short form of MALP2 named MALP2s, efficiently induces immune activation in mouse and human DCs (8, 10, 11). We have chemically synthesized MALP2s composed of the first six amino acids following Pam2 (*S*-[2,3-bis(palmitoyloxy)propyl]-CGNNDE), which lacks the last eight amino acids of full-length MALP2 (Pam2-CGNNDESNISFKEK) (8). MALP2s and MALP2 similarly induce cytokine production from DCs and upregulate major histocompatibility complex (MHC) class I and maturation marker CD86. Antigen (Ag)-specific cytotoxic T lymphocyte (CTL) expansion is primed by DCs, a process called cross-presentation (12). Generally, cross-presentation is augmented by DC maturation involving (i) upregulation of MHC class I molecules; (ii) upregulation of co-stimulatory molecules, including CD80, CD86, and CD40; (iii) increase in Ag peptide-loading on MHC class I; and (iv) production of cytokines enhancing CTL proliferation/activation (13, 14). Since enhancement of cross-presentation was reported with TLR2 ligands (15–17), we assessed T cell cross-priming activity of MALP2s in the present study. We also investigated antitumor activity of MALP2s in tumor-bearing mouse models.

Antitumor immunotherapy is an effective approach to refractory cancers inapplicable to other standard therapies. To evoke a potent antitumor response, an immunostimulatory adjuvant targeting PRRs would be an optimal agent. PRRs recognize pathogen-associated molecular patterns (PAMPs) commonly conserved in foreign microbes. PRRs also recognize damage-associated molecular patterns (DAMPs) released from dying host cells. PRR signaling initiates innate immunity involving DCs, macrophages, and natural killer (NK) cells, and subsequently activates adaptive immunity including T cells and B cells (18). Since a tumor is autologous and lacks endogenous immunostimulatory signals in most cases, an adjuvant targeting PRR is mandatory to invoke an efficient antitumor response. CTLs play a critical role in tumor eradication. Cross-presentation by DC is an essential process for Ag-specific CTL expansion (12). However, immature DCs have poor cross-presentation ability and must mature to induce potent CTL expansion (13). Thus, to develop an effective antitumor immunotherapy, we need to devise an adjuvant capable of inducing DC maturation.

Programmed cell death-1 (PD-1)/programmed death ligand-1 (PD-L1) blockade therapy has improved clinical outcomes in many types of malignant tumors. However, responders to blockade therapy are few, and more than 70–80% of patients still require relief for the unresponsiveness (19, 20). One of the factors influencing therapeutic efficacy is the pre-existence of tumor-specific CTLs (21). The lack of endogenous DC/CTL-priming stimuli may in part cause the unresponsiveness to PD-1/PD-L1 blockade. Determining a CTL-priming adjuvant to complement PD-1/PD-L1 therapy will thus be needed to improve therapeutic efficacy. Here, we investigated the effectiveness of a combination therapy employing MALP2s and PD-L1 blockade.

## Materials and Methods

### Mice

Wild-type (WT) C57BL/6J mice were purchased from CLEA Japan. *Ticam1^−/−^* mice were made in our laboratory. *Ifnar^−/−^, Myd88^−/−^*, and OT-I mice were kindly provided by Dr. T. Taniguchi (Tokyo University, Tokyo, Japan), Dr. S. Akira (Osaka University, Osaka, Japan), and Dr. N. Ishii (Tohoku University, Sendai, Japan), respectively. All mice were backcrossed >8 times to C57BL/6 background and maintained under specific pathogen-free conditions in the animal faculty of the Hokkaido University Graduate School of Medicine. Animal experiments were performed according to the guidelines set by the animal safety center, Hokkaido University, Japan.

### Cells

Mouse bone marrow-derived DCs (BMDCs) were prepared as described previously (22). CD8α^+^ DCs were isolated from mouse spleen by CD8^+^ DC isolation kit (Miltenyi Biotec, the catalog number: 130-091-169). Cells were cultured in RPMI 1640 (GIBCO, 11875-093) supplemented with 10% heat-inactivated FBS (Thermo Scientific, SH30910.03), 10 mM HEPES (GIBCO, 15630-080), and 50 IU penicillin/50 μg/ml streptomycin (GIBCO, 15070-063). EG7 (ATCC^®^ CRL-2113™) cells were purchased from ATCC (Manassas, VA, USA) and cultured in RPMI 1640 supplemented with 10% heat-inactivated FBS, 10 mM HEPES, 1 mM sodium pyruvate (GIBCO, 11360-070), 55 µM 2-mercaptoethanol (GIBCO, 21985-023), 50 IU penicillin/50 μg/ml streptomycin, and 0.5 mg/ml G418 (Roche, 04 727 894 001). MO5 (23) was kindly provided by Dr. H. Udono (Okayama University, Japan) and cultured in RPMI 1640 supplemented with 10% heat-inactivated FBS, 50 IU penicillin/50 μg/ml streptomycin, and 0.1 mg/ml G418.

### Reagents and Antibodies

MALP2s (Pam2CGNNDE) was synthesized by Synpeptide Co., Ltd. (Shanghai, China). Pam2CSK and Pam2CSK4 (Pam2CSKKKK) were synthesized by Biologica Co., Ltd. (Nagoya, Japan). Polyinosinic-polycytidylic acid [poly(I:C)] (27-4732-01) was purchased from GE healthcare Life Sciences. EndogGade^®^ Ovalbumin (OVA) (321001) was purchased from Hyglos. OVA (H2Kb-SL8) Tetramer (TS-5001-P) was purchased from MBL. Mouse interferon (IFN) gamma ELISA KIT (88–7314) was purchased from eBioscience. Carboxyfluorescein diacetate succinimidyl ester (CFSE) (C1157) and Ovalbumin Alexa Fluor™ 647 Conjugate (O34784) were purchased from Molecular Probes. α-PD-L1 antibody (Ab) (clone: 10F.9G2, the catalog number: BE0101) and rat IgG2b isotype control Ab (LTF-2, BE0090) were purchased from Bio X Cell. α-IFNAR-1 Ab (MAR1-5A3, 127304) and mouse IgG1κ isotype control Ab (MOPC-21, 400124) were purchased from BioLegend. Abs used for flow cytometry analysis are listed in Table S1 in Supplementary Material.

### OT-I Proliferation Assay

OT-I T cells were isolated from spleens of OT-I mice by CD8-microbeads (Miltenyi Biotec, 130-049-401). OT-I cells were labeled with 1 µM of CFSE for 10 min at 37°C. In the coculture with OT-I cells and BMDCs, 5 × 10^5^ BMDCs were seeded in a 24-well plate. PBS, 100 nM of Pam2CSK, Pam2CSK4, or MALP2s was added in the wells. After 18 h, 500 ng/ml of OVA was added. After 4 h, OVA was washed out and 1 × 10^5^ BMDCs were re-seeded in a 96-well plate and were cocultured with 1 × 10^5^ CFSE-labeled OT-I cells for 60 h. In the coculture with OT-I cells and CD8α^+^ DCs, 3.5 × 10^4^ CD8α^+^ DCs were seeded in a 96-well plate. PBS, Pam2CSK4, or MALP2s was added in the presence or absence of 2.5 µg/ml of OVA. After 3 h, CD8α^+^ DCs were cocultured with 3.5 × 10^4^ CFSE-labeled OT-I cells for 60 h. After the coculture, cells were stained with anti-CD8α and anti-TCR Vβ5.1,5.2 Abs. Dead cells were excluded by 7-amino actinomycin D staining. OT-I proliferation was evaluated by the attenuation of CFSE with FACS calibur (BD Biosciences). The concentrations of IFN-γ in the culture media were measured by ELISA. For *in vivo* OT-I assay, 6 × 10^5^ CFSE-labeled OT-I cells were intravenously (i.v.) injected to mice. After 24 h, PBS, 25 µg of OVA, or 50 nmol of MALP2s + OVA was subcutaneously (s.c.) injected, respectively. After 60 h, spleens were harvested and OT-I proliferation was evaluated with FACS AriaII (BD Biosciences).

### Tumor Challenge and MALP2s Therapy

Mice were shaved at the back and s.c. injected with 200 µl of 2 × 10^6^ EG7 cells or MO5 cells. Tumor volume was calculated by using the formula: tumor volume [mm^3^] = 0.52 × (long diameter [mm]) × (short diameter [mm])^2^. In the EG7 tumor-bearing model, PBS, 100 µg of OVA, 50 nmol of MALP2s, or MALP2s + OVA was s.c. injected around tumor when the tumor volume reached to 500–600 mm^3^. These treatments were performed once or twice. The second treatment was performed 8 days after the first treatment. For the CD8β^+^ cells or NK1.1^+^ cells depletion, hybridoma ascites containing anti-CD8β or anti-NK1.1 monoclonal Ab was intraperitoneally (i.p.) injected into mice 1 day before MALP2s + OVA treatment. In the MO5 tumor-bearing model, PBS or MALP2s + OVA was s.c. injected around tumor 7 days after tumor implantation. 130 µg of isotype control Ab or α-PD-L1 Ab was i.p. injected into mice on days 7, 9, and 11. Mice were euthanized when a tumor volume reached to 2,500 mm^3^.

### Analysis of Tumor Microenvironment

For a gene expression analysis, a small piece of EG7 or MO5 tumor tissue was collected and total RNA was extracted using Trizol reagent (Thermo Fisher Scientific, 15596-018) as following the manufacturer’s instructions. Real-time PCR was performed as described previously (24). Sequences of primers in this study are shown in Table S2 in Supplementary Material.

For analysis of intratumor CD8^+^ T cells, tumor tissues were finely minced and treated with 0.05 mg/ml collagenase I (Sigma-Aldrich, C0130-100MG), 0.05 mg/ml collagenase IV (Sigma-Aldrich, C5138-1G), 0.025 mg/ml hyaluronidase (Sigma-Aldrich, H6254-500MG), and 0.01 mg/ml DNase I (Roche, 10 104 159 001) in Hank’s Balanced Salt Solution (Sigma-Aldrich, H9269-500ML) at room temperature for 15 min. Tumor-infiltrating CD8^+^ T cells were analyzed by FACS AriaII.

### Statistical Analysis

*p-*Values were calculated by the following statistical analysis. For the multiple comparisons, one-way analysis of variance with Bonferroni’s test or Kruskal–Wallis test with Dunn’s multiple comparison was performed. For the comparison between two groups, Student’s *t*-test was performed. Error bar represent the SD or SEM between samples.

## Results

### MALP2s Induces Ag-Specific CTL Expansion by Augmenting Cross-Presentation of DCs

**Figure 1:**
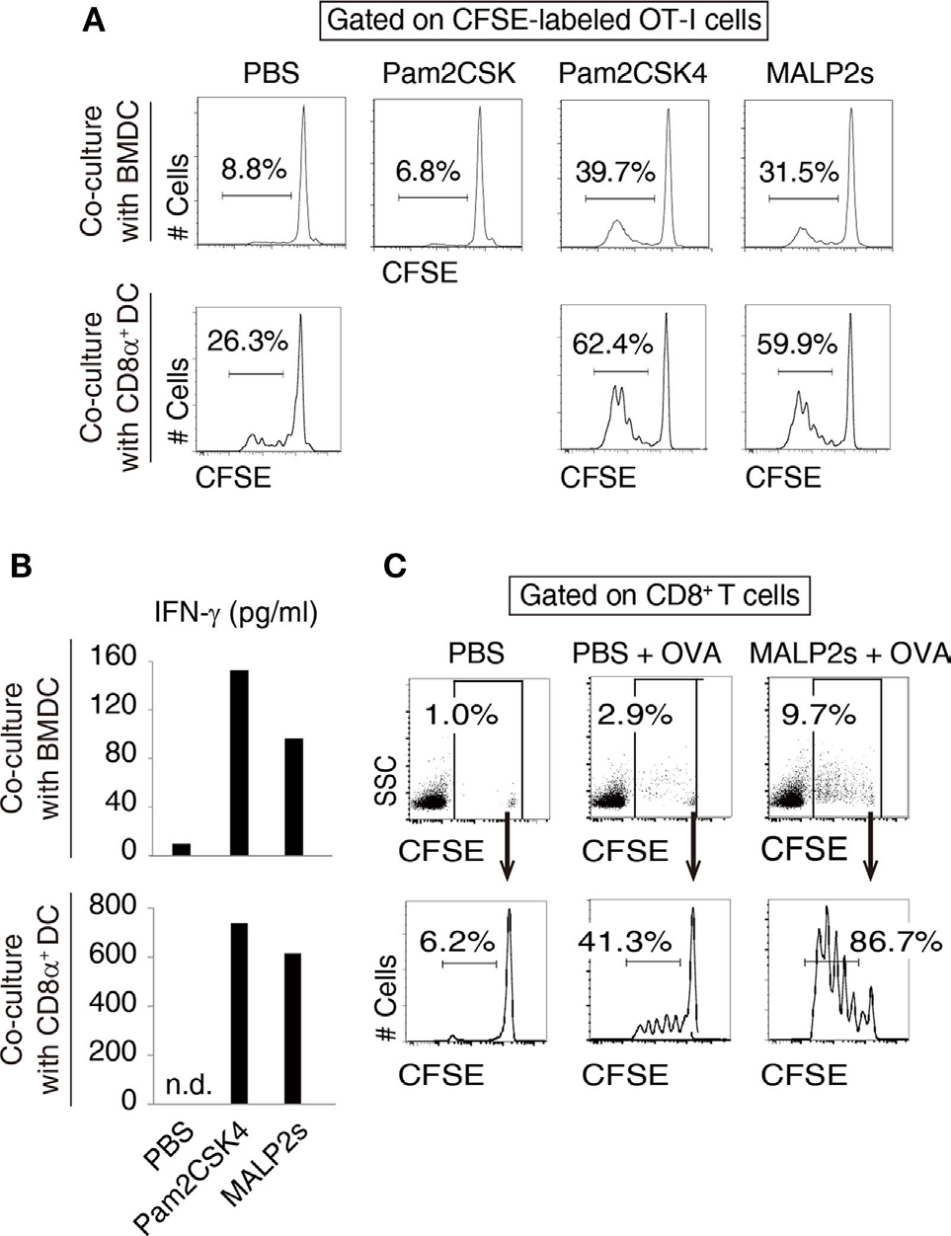
MALP2s induces cross-presentation in dendritic cells (DCs). **(A,B)** Bone marrow-derived dendritic cells (BMDCs) or CD8α^+^ DCs was stimulated with Pam2CSK4 or MALP2s in the presence of ovalbumin (OVA) and cocultured with carboxyfluorescein diacetate succinimidyl ester (CFSE)-labeled OT-I cells. PBS and Pam2CSK were negative controls. **(A)** The percentage of dividing cells among CFSE-labeled OT-I cells was analyzed by flow cytometry. The upper and lower panels show the result of coculture with BMDCs and CD8α^+^ DCs, respectively. **(B)** The concentrations of INF-γ in the culture medium were measured by ELISA. The upper and lower graphs show the results of coculture with BMDCs and CD8α^+^ DCs, respectively. n.d.: not detected. **(C)** CFSE-labeled OT-I cells-transferred WT mice were administered with PBS, OVA, or MALP2s + OVA. The percentage of CFSE-labeled OT-I cells among splenic CD8^+^ T cells (upper panels) and the percentage of dividing cells among CFSE-labeled OT-I cells (lower panels) were analyzed by flow cytometry. *n* = 1 per group. [**(A)** lower graph of **(B)** and **(C)**] The results are representative of more than two independent experiments.

We previously showed that MALP2s upregulated MHC class I and co-stimulatory molecules on mouse BMDCs (8). These responses are the signatures of DC maturation and facilitate Ag-specific CTL priming (25). First, we assessed CTL-priming activity of MALP2s by OT-I proliferation assay. In cocultures of BMDCs and OT-I cells, MALP2s-stimulated BMDCs exhibited higher cross-presentation ability than PBS- or Pam2CSK-added BMDCs in the presence of OVA Ag. Pam2CSK has no TLR2 agonistic activity (26) and was set as a negative control lipopeptide. The degree of OT-I expansion by MALP2s was comparable to Pam2CSK4 (Pam2-CSKKKK), which is another TLR2/6 agonist that exerts DC maturational activity (26) (upper panels of Figure 1A). Since CD8α^+^ DCs are the DC subset which largely contributes to TLR2-induced cross-presentation (15), OT-I proliferation by MALP2s-stimulated CD8α^+^ DCs was also assessed. MALP2s enhanced cross-presentation ability of CD8α^+^ DCs as well as BMDCs (lower panels of Figure 1A). OT-I cells primed by MALP2s- or Pam2CSK4-stimulated BMDCs and CD8α^+^ DCs secreted IFN-γ (Figure 1B; Figure S1A in Supplementary Material). We then performed OT-I proliferation assays *in vivo*. WT mice were injected s.c. with PBS, OVA, or MALP2s + OVA after adoptive transfer of CFSE-labeled OT-I cells. OVA administration but not PBS induced moderate OT-I cell expansion/proliferation without MAPL2s. However, OT-I cell expansion/proliferation was strongly enhanced by co-administration of MALP2s (Figure 1C; upper panels of Figure S1B in Supplementary Material). Moreover, OT-I cells were proliferated in response to i.v. administration of MALP2 + OVA (lower panels of Figure S1B in Supplementary Material). These results suggest that MALP2s is a potent CTL-priming adjuvant.

### The TICAM-1-Type I IFN Axis Does Not Influence MALP2s-Induced DC Maturation

**Figure 2:**
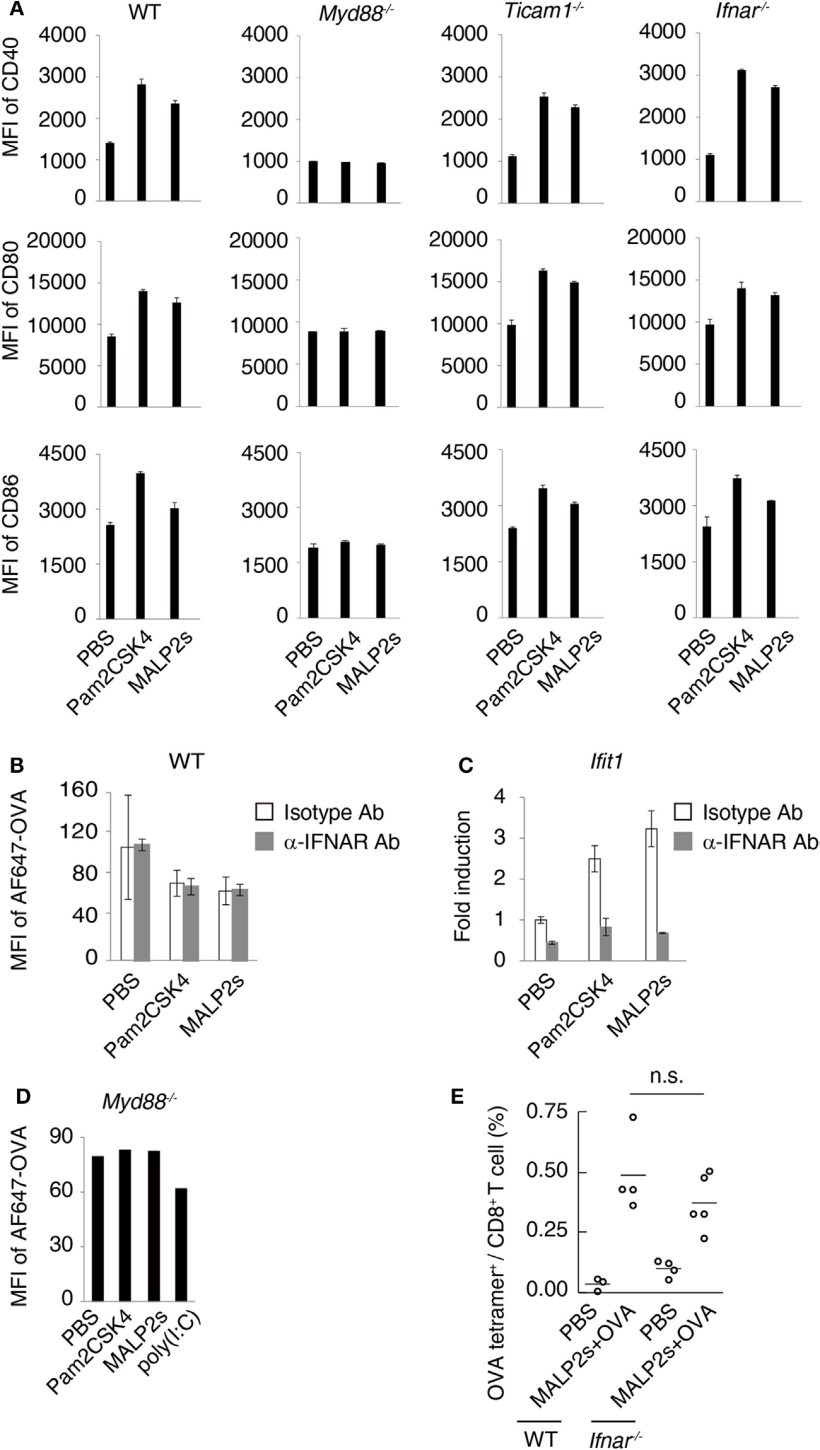
Myeloid differentiation primary response 88 (MyD88) but not TICAM-1-Type I interferon (IFN) pathway is responsible for MALP2s-induced dendritic cell (DC) maturation. **(A)** PBS, 100 nM of Pam2CSK4, or MALP2s was added in bone marrow-derived dendritic cells (BMDCs) derived from various gene knockout mice. After 18 h, CD40, CD80, and CD86 expression levels on DCs were analyzed by flow cytometry. **(B,C)** WT-derived BMDCs were pretreated by 10 µg/ml of isotype antibody (Ab) or α-IFNAR Ab. After 1 h, PBS, Pam2sCSK4, or MALP2s was added. **(B)** After 18 h, 10 µg/ml of AF647-OVA was added and DCs were incubated for 20 min. After washing out OVA, an endocytic activity was assessed by MFI of endocytosed AF647-OVA on BMDCs. **(C)** After 6 h, *Ifit1* gene expression was analyzed by qPCR. **(D)** An endocytic activity of *Myd88^−/−^*-derived BMDCs was assessed as in **(B)**. poly(I:C) was a positive control. **(E)** WT and *Ifnar^−/−^* mice were subcutaneously (s.c.) administered with PBS or MALP2s + OVA. After 7 days, the percentage of OVA-specific cells among splenic CD8^+^ T cells was analyzed by flow cytometry. **(A–C)** Error bars show ± SD. **(E)** *n* = 3 to 5 per group. Kruskal–Wallis test with Dunn’s multiple comparison test was performed to analyze statistical significance. n.s., not significant.

Myeloid differentiation primary response 88 (MyD88) is an adaptor molecule of TLRs including TLR2 but not TLR3. Following ligand recognition by TLR2, nuclear factor-kappa B (NF-κB) and activator protein-1 (AP-1) translocate to the nucleus (5). The activation of transcriptional factors is initiated by MyD88 signaling, which in turn forwards inflammatory responses. Although the MyD88-NF-κB/AP-1 axis does not induce type I IFN, endosomal TLR2 signaling may slightly promote type I IFN production (5, 27–29). Toll-IL-1R homology domain-containing adaptor molecule-1 (TICAM-1, also called TRIF) is the adaptor molecule for TLR3 and TLR4 (30, 31). Nilsen et al. showed that TICAM-1 participates in TLR2-dependent type I IFN production in mouse bone marrow-derived macrophages (29). To assess the contribution of TICAM-1 and type I IFN to TLR2-dependent DC maturation, we evaluated the expression level of maturation markers on WT, *Myd88^−/−^, Ticam1^−/−^*, and *Ifnar^−/−^* BMDCs after MALP2s stimulation. CD40, CD80, and CD86 expression was upregulated by Pam2CSK4 or MALP2s independent of TICAM-1 or IFNAR signaling. The upregulation was not induced at all in *Myd88^−/−^* BMDCs (Figure 2A). Since a decrease of endocytosis/phagocytosis is one of the signatures of DC maturation (32), endocytic activity in MALP2s-stimulated BMDCs was also evaluated. IFNAR signaling blockage by α-IFNAR Ab treatment did not affect the decrease in endocytic activity of DCs induced by Pam2CSK4 and MALP2s (Figure 2B). In this setting, α-IFNAR Ab treatment completely blocked induction of the IFN-inducible gene *Ifit1* by TLR2 ligands (Figure 2C). The endocytic activity of *Myd88^−/−^* BMDCs was also evaluated. The TLR3 agonist poly(I:C) was set as a positive control because TLR3-induced DC maturation is independent of MyD88. The endocytic activity of *Myd88^−/−^* BMDCs was decreased by poly(I:C) but not by TLR2 ligands (Figure 2D). These results indicate that TLR2-induced DC maturation completely depends on the MyD88 pathway: MyD88-derived DC priming exists independent of TICAM-1 and type I IFN signaling.

We further analyzed the contribution of type I IFN-IFNAR signaling in MALP2s-induced cross-presentation by tetramer assay. *Ifnar^−/−^* mice immunized with MALP2s and OVA showed the expansion of OVA-specific CD8^+^ T cells in the spleen at the same rate as observed in WT mice (Figure 2E). The result indicates that MALP2s induces Ag-specific CTLs irrespective of the type I IFN signal.

### MALP2s/TAA Therapy Strongly Regresses CTL-Susceptible T Cell Lymphoma EG7

**Figure 3:**
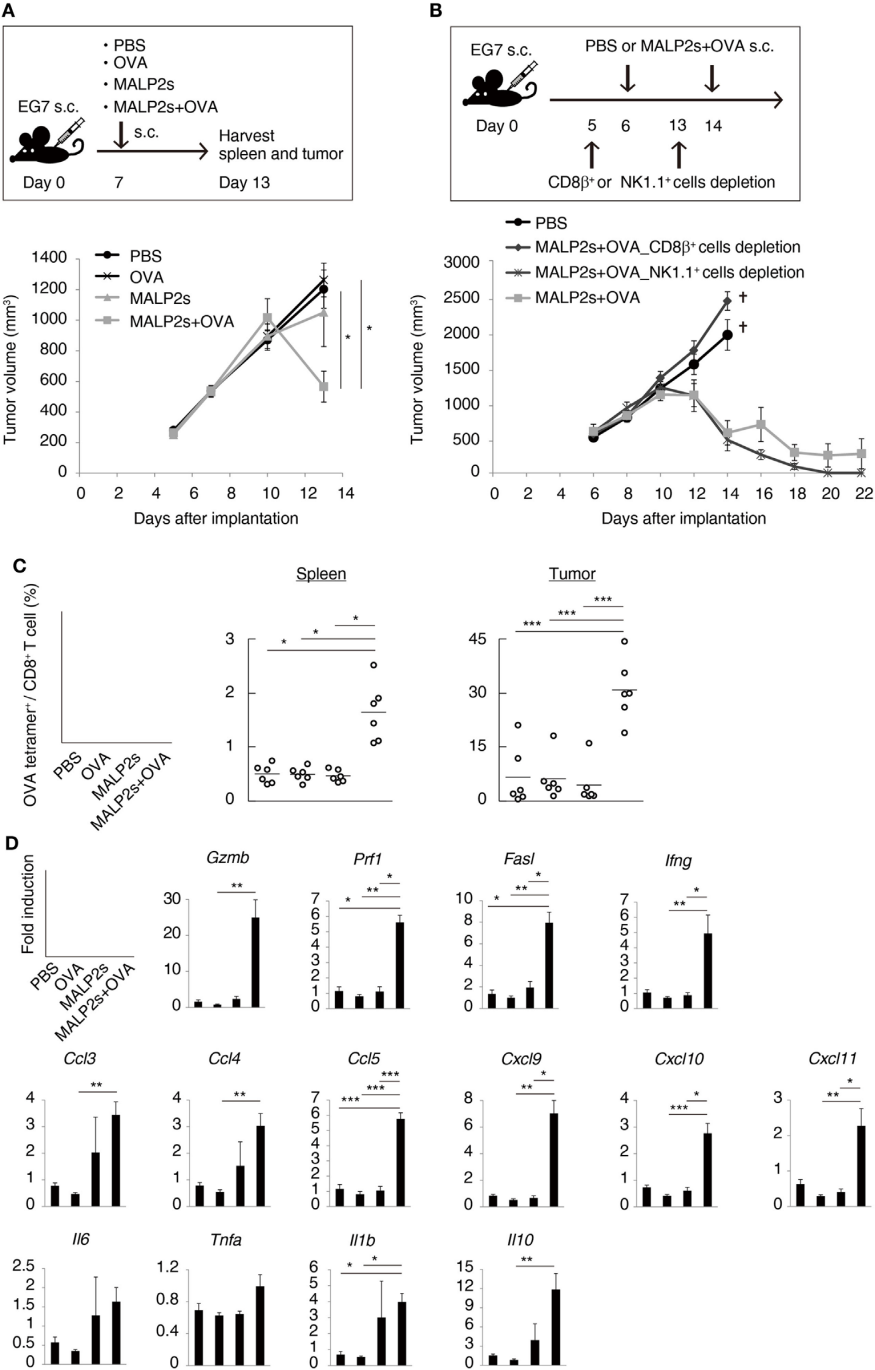
MALP2s with tumor-associated antigen regresses EG7 tumor in a cytotoxic T lymphocytes-dependent manner. **(A,B)** EG7-bearing mice were treated as in the upper schemes. A tumor volume was measured in each group (lower Figures). PBS and CD8β^+^ cells depletion groups of B were euthanized on day 14. **(C,D)** EG7-bearing mice of A were euthanized on day 13. **(C)** The percentages of OVA-specific cells among splenic and intratumor CD8^+^ T cells were analyzed by flow cytometry. **(D)** Gene expressions in tumor tissue were analyzed by qPCR. Error bars show ± SEM; *n* = 4–6 per group. Kruskal–Wallis test with Dunn’s multiple comparison test or One-way analysis of variance with Bonferroni’s test was performed to analyze statistical significance (*p < 0.05, **p < 0.01 ***p < 0.001). **(A,C)** The results are representative of more than two independent experiments.

Tumor-associated antigen (TAA)-specific CTL plays an important role in effective cancer immunotherapy. We evaluated the potential of MALP2s as an antitumor adjuvant in a tumor-bearing mouse model. EG7 (OVA-positive EL4 T cell lymphoma)-implanted mice were locally administered with PBS, OVA, MALP2s, or MALP2s + OVA. Although OVA or MALP2s administration did not suppress tumor growth, the combination with MALP2s and OVA exerted potent tumor regressive effects (Figure 3A). The MALP2s/OVA-induced tumoricidal effect was completely dependent on CTL, while NK cells did not contribute to tumor regression (Figure 3B). On the sixth day after the MALP2s/OVA treatment, OVA-specific CD8^+^ T cells had expanded in the spleen and infiltrated the tumor tissue (Figure 3C). The OVA-specific CD8^+^ T cell induction was not observed in the PBS, OVA, and MALP2s groups (Figure 3C). Gene expression in tumor tissue was also analyzed simultaneously. The genes related to CTL cytotoxicity (*Gzmb, Prf1, Fasl*, and *Ifng*) and the chemokine genes related to CTL recruitment (*Ccl3, 4*, and *5*; *Cxcl9, 10*, and *11*) were elevated by MALP2s/OVA treatment (Figure 3D). The inflammatory cytokines (*Il6, Tnfa*, and *Il1b*) and immunosuppressive cytokine (*Il10*) were also analyzed. *Il1b* and *Il10* but not *Il6* and *Tnfa* were significantly elevated by MALP2s/OVA treatment (Figure 3D). These results suggest that the combination therapy of MALP2s and TAA is an effective antitumor strategy in a CTL-susceptible tumor, though the cytokine problem still exists.

### MALP2s and TAA Therapy Enhanced PD-L1 Blockade Efficacy

**Figure 4:**
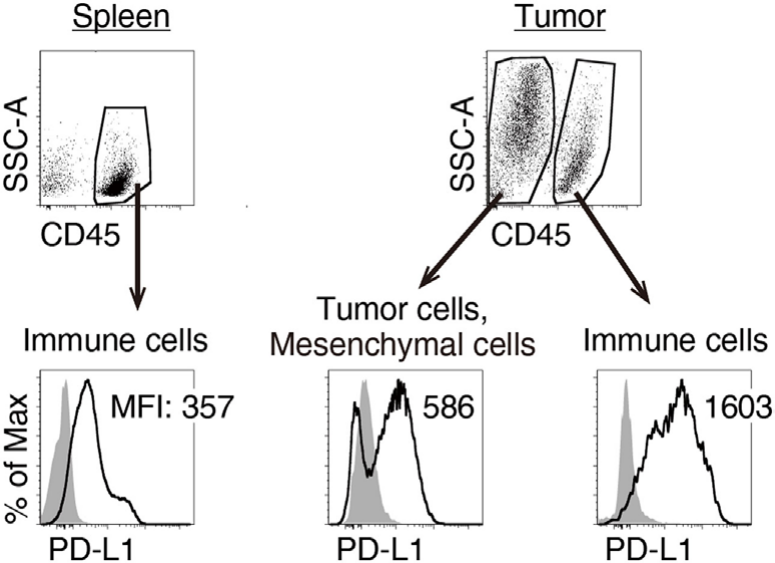
Splenic and intratumor immune cells express programmed death ligand-1 (PD-L1). MO5-bearing mice were euthanized 16 days after tumor implantation. PD-L1 expression levels on splenic CD45^+^ cells, intratumor CD45^−^ and CD45^+^ cells were analyzed by flow cytometry. Shaded and open histograms indicate isotype control and PD-L1 staining, respectively. The results are representative of more than three independent experiments.

**Figure 5:**
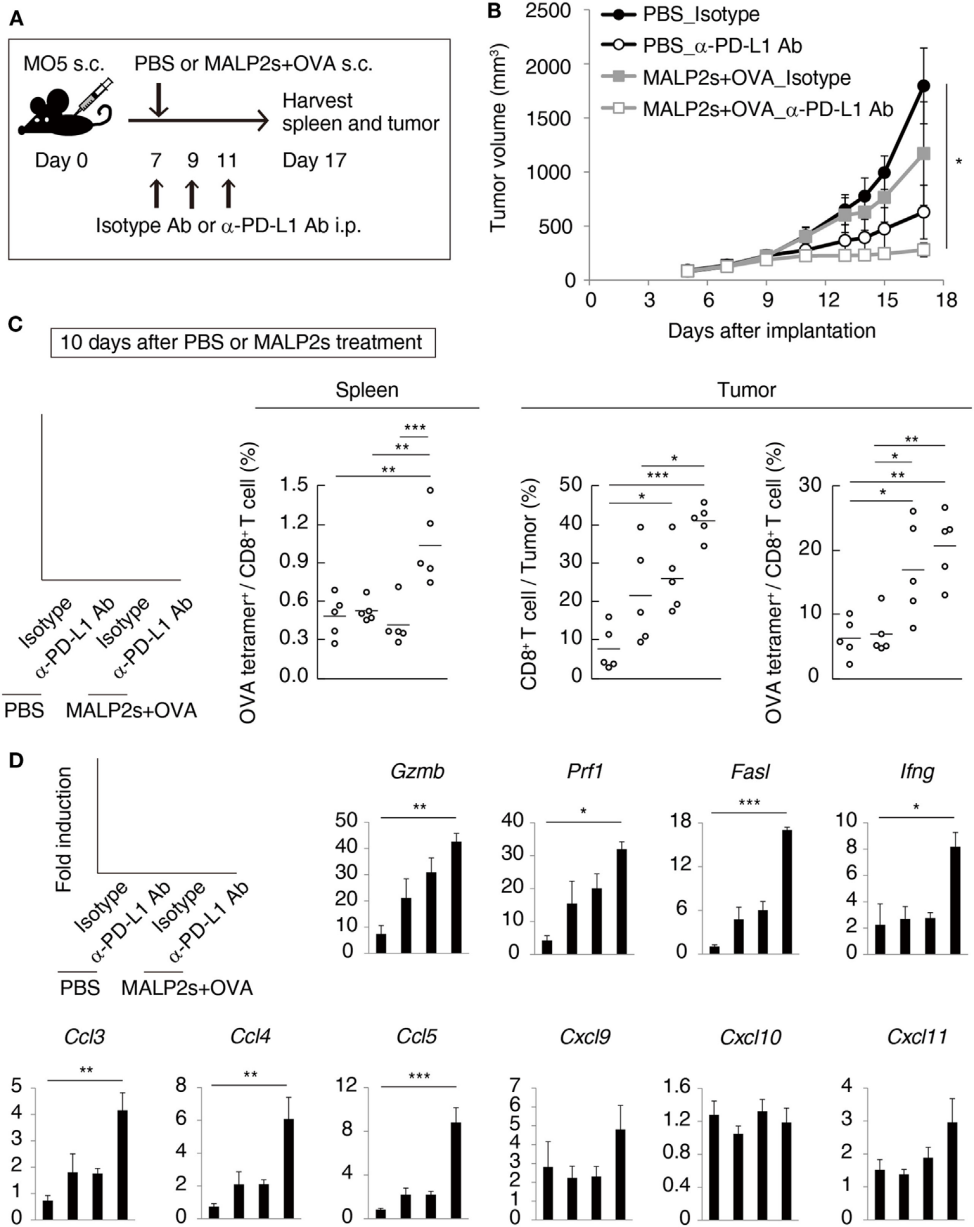
MALP2s with tumor-associated antigen enhances antitumor response by programmed death ligand-1 (PD-L1) blockade. **(A)** The scheme of each treatment on MO5-bearing mice is shown. **(B)** A tumor volume was measured in each group. **(C,D)** Mice were euthanized on day 17. **(C)** The percentages of tumor-infiltrating CD8^+^ T cells and splenic and intratumor OVA-specific CD8^+^ T cells were analyzed by flow cytometry. **(D)** Gene expressions in tumor tissue were analyzed by qPCR. Error bars show ± SEM; *n* = 5 per group. Kruskal–Wallis test with Dunn’s multiple comparison test or One-way analysis of variance with Bonferroni’s test was performed to analyze statistical significance (*p < 0.05, **p < 0.01 ***p < 0.001).

We previously reported that TLR3-specific CTL-priming adjuvant enhanced the therapeutic efficacy of PD-L1 blockade in some tumor-bearing mice models (33). Here, we also investigated the availability of MALP2s as an enhancer of PD-L1 blockade therapy in the MO5 (OVA-positive melanoma)-bearing mouse model. In tumor tissue from the MO5-bearing mouse, PD-L1 molecules expressed not only on the CD45^−^ population including MO5 cells and mesenchymal cells but also on the CD45^+^ population representing intratumor immune cells (Figure 4). The PD-L1 expression level was higher in intratumor immune cells than in MO5 cells. PD-L1 was also expressed in splenic immune cells (Figure 4). The PD-L1 distribution suggests that not only tumor cells but also intratumor and splenic immune cells are targets of PD-L1 blockade. MO5-implanted mice were locally administered with PBS or MALP2s + OVA and also treated with isotype Ab or α-PD-L1 Ab (Figure 5A). Both MALP2s + OVA and α-PD-L1 Ab therapy partially suppressed tumor growth, but the combination of MALP2s/OVA with α-PD-L1 Ab culminated in maximal tumor suppression (Figure 5B). On day 7 after MALP2s/OVA treatment, OVA-specific CD8^+^ T cells had expanded in the spleen and infiltrated tumor tissue even without PD-L1 blockade (Figures S2A,B in Supplementary Material). However, on day 10 after MALP2s/OVA treatment without PD-L1 blockade, splenic OVA-specific CD8^+^ T cells were decreased (Figure 5C). The proportion of OVA-specific cells in splenic CD8^+^ T cells was also low in PBS and α-PD-L1 Ab monotherapy groups. On the other hand, the level of OVA-specific CD8^+^ T cells was maintained high in spleens after combination therapy of MALP2s/OVA with α-PD-L1 Ab (Figure 5C). CD8^+^ T cells infiltrated and remained longer in tumors by MALP2s/OVA therapy than α-PD-L1 Ab monotherapy. The infiltration increased most prominently by the combination therapy with MALP2s/OVA and α-PD-L1 Ab (Figure 5C). The ratio of OVA-specific cells among intratumor CD8^+^ T cells was high in MALP2s/OVA groups with or without PD-L1 blockade, while α-PD-L1 Ab monotherapy did not induce TAA-specific CD8^+^ T cell infiltration (Figure 5C). Gene expression in tumor tissue was also analyzed. Both α-PD-L1 Ab and MALP2s/OVA therapy modestly increased the expression levels of CTL cytotoxicity-related genes, but expression was increased most substantially after combination therapy. Combination therapy also significantly increased the expression of chemokine genes *Ccl3, 4*, and *5* (Figure 5D). These results suggest that the MALP2s and TAA therapy enhance the efficacy of PD-L1 blockade by evoking TAA-specific CTL expansion in lymphoid tissue and facilitating TAA-specific CTL infiltration into the tumor site.

## Discussion

The existence of TAA-specific CTLs is a key factor affecting clinical outcomes in many types of cancer. Many clinical trials have shown that intratumor CD8^+^ T cell levels positively correlate with overall survival and the success of antitumor immunotherapy (34). For the expansion of TAA-specific CTLs, cross-presentation by mature DCs is essential. Thus, an immunostimulatory adjuvant like a TLR agonist evoking DC maturation and subsequent CTL priming may be a promising antitumor agent. Human conventional DCs only express TLR2/TLR1/TLR6 and TLR4, in addition to TLR3 (35). In particular, human CD141^+^ DCs, which are a professional Ag-presenting DC subset, showed selective high expression of TLR3, TLR2/TLR1, and TLR 6 (36). TLR1 and TLR6 are co-receptors of TLR2 for recognition of Pam3 and Pam2 lipopeptides, respectively (9, 37). Hence, the application of a TLR2 agonist as a clinical antitumor adjuvant is rational.

We previously showed that the short length of three amino acids following Pam2Cys was sufficient for TLR2 stimulation. The first amino acid residue following Pam2Cys determined the agonistic activity and Ser/Thr or Gly/Ala were functional residues (26). The first amino acid residue following Pam2Cys of MALP2s is Gly. In this study, MALP2s promoted DC maturation and potent cross-priming activity (Figures 1 and 2). The sequence containing the last eight amino acids of full-length MALP2 was not essential for cross-presentation. Amino acid substitution studies support this idea (38): we have evidence that the peptide sequence of the Pam2 lipopeptide alters the TLR2 agonistic function in DC models (26). The MALP2-induced inflammatory cytokines and NO production fully depend on the MyD88 pathway (39). Recent findings revealed that TLR2 signaling induces not only inflammatory cytokines but also low amounts of type I IFN production (5, 27–29). TLR2 located on the endosomal compartment but not on the plasma membrane can induce type I IFN production. Although the intracellular signaling pathway is still unclear and there seems to be a difference among cell types, the MyD88–interferon regulatory factors axis may be dispensable for amplifying type I IFN production. TICAM-1 and/or TRAM molecules appeared to partially contribute to type I IFN production from bone marrow-derived macrophages (28, 29). Since type I IFN is an inducer of DC maturation and cross-presentation (40), we assessed the contribution of type I IFN to MALP2s-induced CTL priming. While TICAM-1 and type I IFN signaling were not involved in DC maturation, MyD88 was essential for MALP2s-mediated DC priming. Type I IFN signaling was also not required for MALP2s-induced cross-priming (Figure 2). The MyD88–NF-κB/AP-1 axis has been considered as the pathway responsible for CTL priming, but details of the intracellular molecular cascade have not been elucidated. Lymphocytes also utilize the MyD88 pathway (41), but their participation, if any, in antitumor CTL proliferation for tumor regression appears to be minimal, at least in this tumor model.

The antitumor activity of MALP2 has been shown in a pancreatic cancer-bearing mouse model (42). MALP2 administration prolonged survival and enhanced the effectiveness of combination therapy with gemcitabine. We show here that MALP2 induces splenic CD8^+^ T cell expansion. Clinical research on MALP2 administration has been also performed in pancreatic carcinoma patients (43). In phase I/II trials, patients were treated with MALP2 and gemcitabine. Although the number of patients was small and clinical efficacy still remains to be demonstrated, the results suggested the potential of MALP2 using as a clinical antitumor adjuvant. One potential issue, however, was not the DC-priming activity but rather the cytokine toxicity of MALP2. We thus evaluated the antitumor activity of MALP2s in OVA-positive lymphoma (EG7)- and melanoma (MO5)-bearing mouse models. In the EG7-bearing model, MALP2s monotherapy did not show any tumor suppressive activity. However, combination with TAA dramatically induced CTL-dependent tumor regression, facilitated CTL priming in lymphoid tissue and fostered TAA-specific CTL migration to tumor sites (Figure 3). The result indicates that endogenous TAA is insufficient to evoke CTL induction in MALP2s therapy and that co-administration of exogenous TAA is needed. For future clinical application, appropriate selection and supply of exogenous TAA should be established.

The synthesis of a fusion peptide containing MALP2s and a TAA epitope might be a valid strategy. Shen et al. designed Pam2 lipopeptides containing the H-2D^b^-restricted CTL epitope from HPV16 E7 protein and showed it had cross-priming activity (17). The short length of MALP2s may be suitable for the design of a fusion peptide. However, we have no rationale why the fusion of TAA with MALP2 is better than separate MALP2 and TAA administrations (44), since each acts upon different targets in DCs.

Immune checkpoint inhibition including CTL-associated Ag 4 and PD-1/PD-L1 blockade shows great clinical efficacy in refractory and metastatic cancers. However, the number of patients responsive to blockade therapy is small. A valid therapeutic strategy to overcome this clinical limitation is strongly desired. Neoantigen load may be a useful biomarker for assessing the sensitivity to immune checkpoint blockade which positively correlates with clinical benefit (45–48). However, nonresponders to blockade therapy with high neoantigen loads also exist (45, 47, 49). This fact suggests that even if TAA has immunogenicity, TAA peptide administration alone is insufficient and an additional factor may be necessary to evoke an antitumor response in some unresponsive patients. Roony et al. showed that not only neoantigen load but also the existence of an endogenous or exogenous virus was positively correlated with a cytolytic activity against tumors (50). DAMP might positively or negatively regulate the tumor microenvironment in PD-1/PD-L1 therapy. However, the exact constitution of DAMPs has not yet been identified. In contrast, the PAMPs which trigger cross-presentation with defined molecules are supported by protein–chemical database background. We previously showed that EG7, which expresses immunogenic OVA, was unresponsive to α-PD-L1 Ab monotherapy and that combination therapy with a TLR3 agonist relieved the unresponsiveness (33). Intratumor CD8^+^ T cells were few in EG7-bearing mice with or without α-PD-L1 Ab monotherapy, and this observation suggests that EG7 lacks an endogenous immune stimulator like DAMPs. In addition to TLR3 agonists, TLR7 and TLR9 agonists also augmented the therapeutic efficacy of PD-1/PD-L1 blockade in preclinical mouse models (51, 52). These findings suggest that TLR adjuvant with DC-priming activity may overcome the clinical limitation of immune checkpoint blockade in patients lacking an endogenous immune stimulator. However, circumvention of cytokine toxicity still remains problematic. How we should design a less toxic derivative based on our current knowledge of the Pam2 lipopeptide (26) is an issue that will soon need to be addressed.

While TLR2 agonists have antitumor activity, tumor-supportive activities have also reported. TLR2 signaling promoted survival and proliferation of certain types of tumors (53). TLR2 signaling also promoted expansion of myeloid-derived suppressor cells and regulatory T cell expansion and enhanced their suppressive functions (54, 55). In order to eliminate or reduce these tumor-supportive effects, DC targeting by adjuvant, particularly DC-priming adjuvant, may be effective. Akazawa et al. designed a Pam2 lipopeptide containing a DC-targeting sequence which showed antitumor activity (56). MALP2s will likely be an appropriate lipopeptide for sequence modulation that can expect further development.

In conclusion, we showed the synergistic antitumor effect of MALP2s/TAA and α-PD-L1 Ab treatment (Figure 5). MALP2s/TAA contributed to TAA-specific CTL priming in lymphoid tissue while α-PD-L1 Ab helped to prevent CTL exhaustion and cell death in both lymphoid tissue and tumor tissue. Molecular modification of MALP2s will be a strategy for further development of an antitumor adjuvant that overcomes PD-1/PD-L1 blockade resistance in patients.

## Ethics Statement

All animal research protocols for this work were reviewed and approved by the Animal Safety Center (#17-0096) in Hokkaido University, Japan.

## Author Contributions

YT, MA, KF, MM, and TS conceived and designed the experiments. YT, MA, and KF performed the experiments. YT, MA, KF, HS, MM, and TS analyzed the data. YT, MM, and TS wrote the paper. MM and TS supervised the research.

## Conflict of Interest Statement

The authors declare that the research was conducted in the absence of any commercial or financial relationships that could be construed as a potential conflict of interest. The reviewer DO and handling editor declared their shared affiliation.

## Abbreviations

Ab, antibody; Ag, antigen; AP-1, activator protein-1; BMDC, bone marrow-derived dendritic cell; CFSE, carboxyfluorescein diacetate succinimidyl ester; CTLs, cytotoxic T lymphocytes; DAMPs, damage-associated molecular patterns; DC, dendritic cell; IFN, interferon; i.p., intraperitoneally; i.v., intravenously; MALP2, macrophage-activating lipopeptide 2; MHC, major histocompatibility complex; MyD88, myeloid differentiation primary response 88; NF-κB, nuclear factor-kappa B; OVA, ovalbumin; PAMPs, pathogen-associated molecular patterns; PD-1, programmed cell death-1; PD-L1, programmed death ligand-1; poly(I:C), polyinosinic-polycytidylic acid; PRR, pattern-recognition receptors; s.c., subcutaneously; TAA, tumor-associated antigen; TICAM-1, toll-IL-1R homology domain-containing adaptor molecule-1; TLR, toll-like receptor; WT, wild type.
