# Supplementary material for: Type I Interferon-Independent Dendritic Cell Priming and Antitumor T Cell Activation Induced by a Mycoplasma fermentans Lipopeptide
Source: Front Immunol. 2018 Mar 14;9:496. doi: 10.3389/fimmu.2018.00496 (PMC5861346; doi:10.3389/fimmu.2018.00496)
Supplement: Supplementary file 1 [file data_sheet_1.docx]

***Supplementary Material***

**Type I interferon-independent dendritic cell priming and antitumor T cell activation induced by a *Mycoplasma fermentans* lipopeptide**

Yohei Takeda^*^, Masahiro Azuma, Kenji Funami, Hiroaki Shime, Misako Matsumoto and Tsukasa Seya^*^.

*** Correspondence**

Tsukasa Seya [seya-tu@pop.med.hokudai.ac.jp](mailto:seya-tu@pop.med.hokudai.ac.jp)

Yohei Takeda [ykyyou@med.hokudai.ac.jp](mailto:ykyyou@med.hokudai.ac.jp)

**
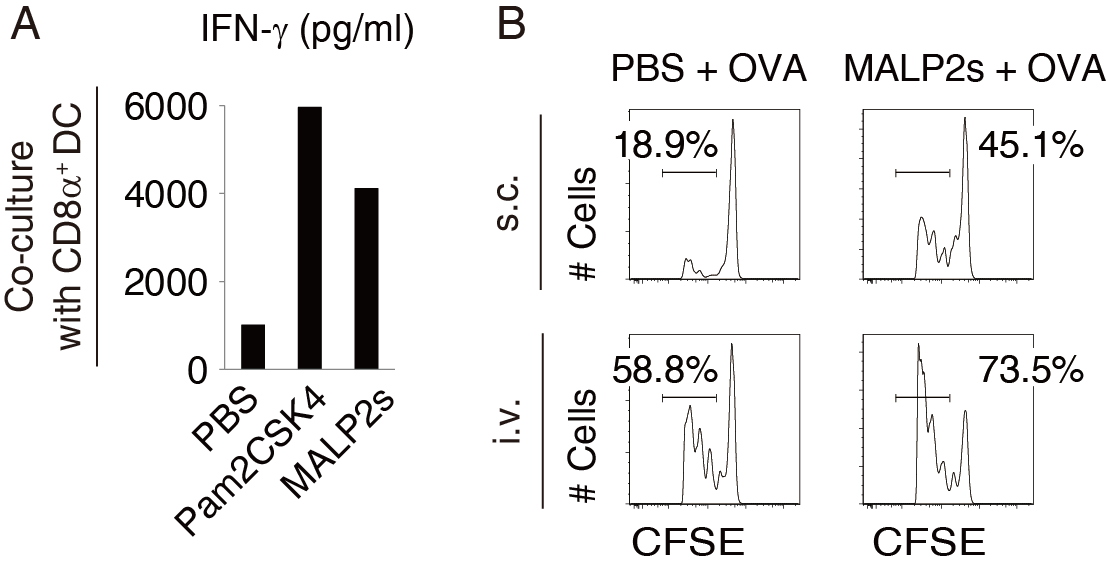
**

**Supplemental Figure 1.** The addendum data related to Figure 1B and C. **(A)** The second experiment similar to lower graph of Figure 1B. **(B)** The second experiment similar to Figure 1C (upper panels). CFSE-labeled OT-I cells-transferred WT mice were i.v. administered with 5 μg of OVA or 50 nmol of MALP2s+OVA. The percentage of dividing cells among CFSE-labeled OT-I cells was shown (lower panels). n = 1 per group.

**
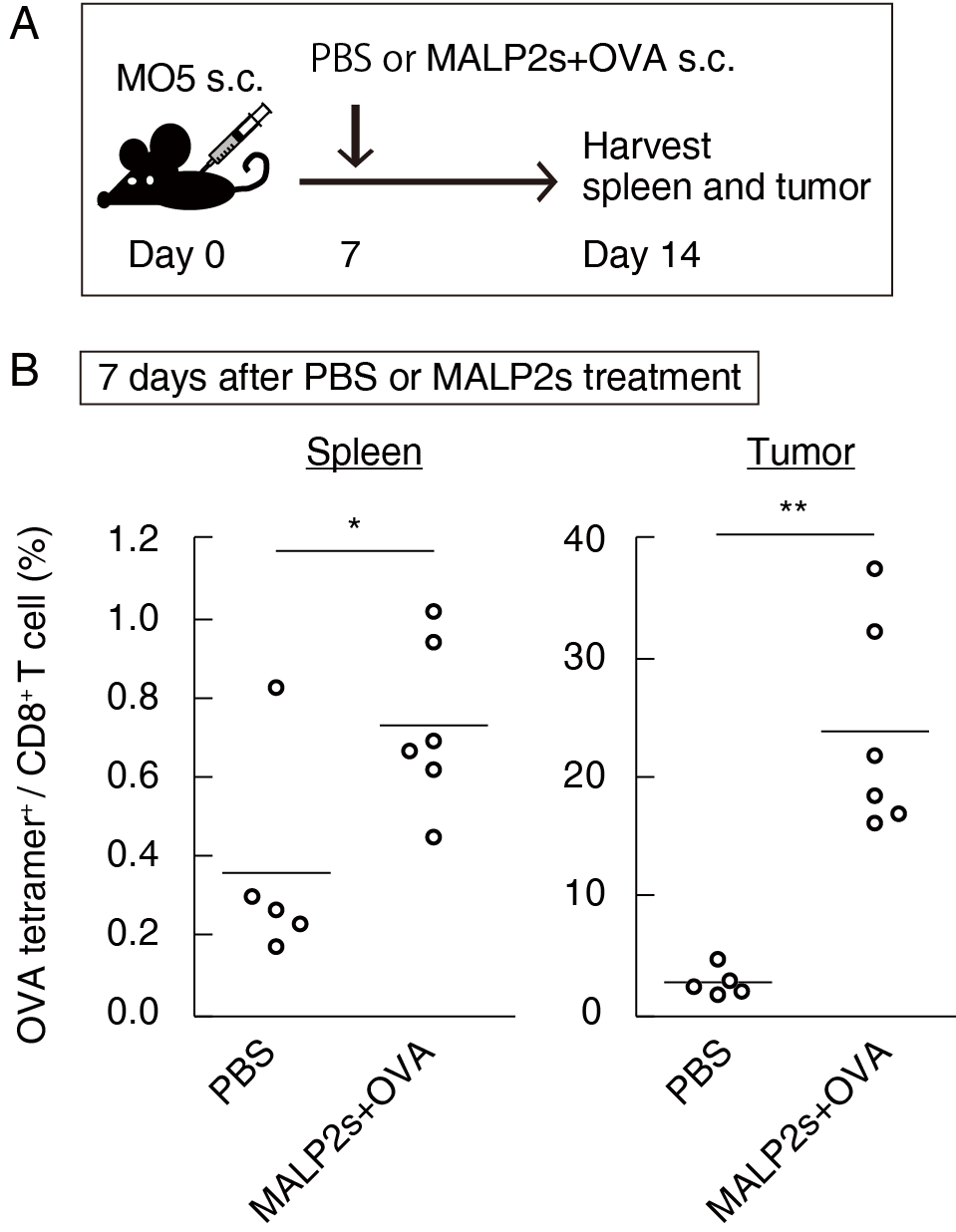
**

**Supplemental Figure 2.** MALP2s with TAA induces Ag-specific CD8^+^ T cells in early phase. Related to Figure 5. **(A)** The scheme of each treatment on MO5-bearing mice is shown. **(B)** Mice were euthanized on day 14. The percentages of OVA-specific cells among splenic and intratumor CD8^+^ T cells were analyzed by flow cytometry. Error bars show ± SEM; n = 5 to 6 per group. Student’s t-test was performed to analyze statistical significance.*p < 0.05, **p < 0.01. The results are representative of more than two independent experiments.

**Supplemental Table 1.** Antibodies used for flow cytometry analysis.

| Antibody | Clone | Catalog No. | Supplier |
| --- | --- | --- | --- |
| Alexa Fluor700-α-mouse CD3  Pe/Cy7-α-mouse CD3 | 17A2 | 100216  100220 | Biolegend |
| Alexa Fluor700-α-mouse CD8α  APC-α-mouse CD8α | 53-6.7 | 100730  100712 |  |
| APC-α-mouse CD11c | N418 | 117310 |  |
| α-mouse CD16/32 | 93 | 101302 |  |
| FITC-α-mouse CD40 | 3/23 | 124607 |  |
| Alexa Fluor700-α-mouse CD45.2 | 104 | 109822 |  |
| PE-α-mouse CD80 | 16-10A1 | 104707 |  |
| FITC-α-mouse CD86 | GL-1 | 105005 |  |
| PE- α-TCR Vβ5.1,5.2 | MR9-4 | 139504 |  |
| PE-α-mouse PD-L1 | MIH5 | 12-5982-82 | eBioscience |
| ViaProbe (PerCP/Cy5.5-7AAD) |  | 555816 | BD Biosciences |

| **Gene** | **Primer sequences** | |
| --- | --- | --- |
|  | **Forward** | **Reverse** |
| *Gapdh* | 5’-GCCTGGAGAAACCTGCCA-3’ | 5’-CCCTCAGATGCCTGCTTCA-3’ |
| *Ccl3* | 5’-TTGAAACCAGCAGCCTTTGC-3’ | 5’-CTTTGGAGTCAGCGCAGATCT-3’ |
| *Ccl4* | 5’-GCCCTCTCTCTCCTCTTGCT-3’ | 5’-GGAGGGTCAGAGCCCATT-3’ |
| *Ccl5* | 5’-TGCCCACGTCAAGGAGTATTT-3’ | 5’-TCGAGTGACAAACACGACTGC-3’ |
| *Cxcl9* | 5’-GATAAGGAATGCACGATGCTC-3’ | 5’-TCTCCGTTCTTCAGTGTAGCAA-3’ |
| *Cxcl10* | 5’-GTGTTGAGATCATTGCCACGA-3’ | 5’-GCGTGGCTTCACTCCAGTTAA-3’ |
| *Cxcl11* | 5’-GGCTGCGACAAAGTTGAAGTGA-3’ | 5’-TCCTGGCACAGAGTTCTTATTGGAG-3’ |
| *Fasl* | 5’-TTAAATGGGCCACACTCCTC-3’ | 5’-ACTCCGTGAGTTCACCAACC-3’ |
| *Gzmb* | 5’-TCCTGCTACTGCTGACCTTGTC-3’ | 5’-ATGATCTCCCCTGCCTTTGTC-3’ |
| *Ifit1* | 5’-TGTGCTGAGATGGACTGTGAG-3’ | 5’-TTTCTGGCTCCACTTTCAGAG-3’ |
| *Ifng* | 5’-GATATCTGGAGGAACTGGCAAAAG-3’ | 5’-AGAGATAATCTGGCTCTGCAGGAT-3’ |
| *Il1b* | 5’-TGACGGACCCCAAAAGATGA-3’ | 5’-TGCTGCTGCGAGATTTGAAG-3’ |
| *Il6* | 5’-GTTCTCTGGGAAATCGTGGA-3’ | 5’-TCCAGTTTGGTAGCATCCATC-3’ |
| *Il10* | 5’-GGCGCTGTCATCGATTTCTC-3’ | 5’-TGCTCCACTGCCTTGCTCTTA-3’ |
| *Prf1* | 5’-CAAGGTAGCCAATTTTGCAGC-3’ | 5’-GGCGAAAACTGTACATGCGAC-3’ |
| *Tnfa* | 5’-AATGGCCTCCCTCTCATCAGT-3’ | 5’-GCTACAGGCTTGTCACTCGAATT-3’ |

**Supplemental Table 2.** Primer sequences used for real-time RT-PCR.
